# Local anesthetics impair the growth and self-renewal of glioblastoma stem cells by inhibiting ZDHHC15-mediated GP130 palmitoylation

**DOI:** 10.1186/s13287-021-02175-2

**Published:** 2021-02-04

**Authors:** Xiaoqing Fan, Haoran Yang, Chenggang Zhao, Lizhu Hu, Delong Wang, Ruiting Wang, Zhiyou Fang, Xueran Chen

**Affiliations:** 1 Department of Anesthesiology, Division of Life Sciences and Medicine, The First Affiliated Hospital of USTC, University of Science and Technology of China (USTC), No. 17, Lujiang Road, Hefei, 230001 Anhui China; 2 Department of Medical Laboratory, Hefei Cancer Hospital, Chinese Academy of Sciences, No. 350, Shushan Hu Road, Hefei, 230031 Anhui China; 3 Anhui Province Key Laboratory of Medical Physics and Technology, Institute of Health and Medical Technology, Hefei Institutes of Physical Science, Chinese Academy of Sciences, No. 350, Shushan Hu Road, Hefei, 230031 Anhui China

**Keywords:** Local anesthetics, Glioblastoma stem cells, Self-renewal, ZDHHC15, GP130, STAT3

## Abstract

**Background:**

A large number of preclinical studies have shown that local anesthetics have a direct inhibitory effect on tumor biological activities, including cell survival, proliferation, migration, and invasion. There are few studies on the role of local anesthetics in cancer stem cells. This study aimed to determine the possible role of local anesthetics in glioblastoma stem cell (GSC) self-renewal and the underlying molecular mechanisms.

**Methods:**

The effects of local anesthetics in GSCs were investigated through in vitro and in vivo assays (i.e., Cell Counting Kit 8, spheroidal formation assay, double immunofluorescence, western blot, and xenograft model). The acyl-biotin exchange method (ABE) assay was identified proteins that are S-acylated by zinc finger Asp-His-His-Cys-type palmitoyltransferase 15 (ZDHHC15). Western blot, co-immunoprecipitation, and liquid chromatograph mass spectrometer-mass spectrometry assays were used to explore the mechanisms of ZDHHC15 in effects of local anesthetics in GSCs.

**Results:**

In this study, we identified a novel mechanism through which local anesthetics can damage the malignant phenotype of glioma. We found that local anesthetics prilocaine, lidocaine, procaine, and ropivacaine can impair the survival and self-renewal of GSCs, especially the classic glioblastoma subtype. These findings suggest that local anesthetics may weaken ZDHHC15 transcripts and decrease GP130 palmitoylation levels and membrane localization, thus inhibiting the activation of IL-6/STAT3 signaling.

**Conclusions:**

In conclusion, our work emphasizes that ZDHHC15 is a candidate therapeutic target, and local anesthetics are potential therapeutic options for glioblastoma.

**Supplementary Information:**

The online version contains supplementary material available at 10.1186/s13287-021-02175-2.

## Background

Local anesthetics are widely used in clinical cancer surgery [1, 2]. Current data indicate that anesthetics or anesthesia techniques may affect cancer metastasis and postoperative recurrence [3, 4]. When local anesthetics are absorbed into the circulation or administered intravenously, they have a direct effect on cancer cells [3]. Local anesthetics may block voltage-gated sodium channels (VGSC) and cause depolarization of excitable cells [5]. Recent studies have shown that many metastatic cancers also exhibit abnormally high levels of VGSC expression and are closely related to the clinical stage, recurrence, drug resistance, and prognosis of cancer [6]. However, the effect of local anesthetics on the survival rate of cancer patients after surgery is controversial [7–9]. So far, little is known about the direct effect of local anesthetics on cancer cells.

A relatively unexplored area is the effect of local anesthetics on cancer stem cells (CSCs). The existence of glioblastoma (GBM) stem cells (GSCs) raises the question of whether GSCs or differentiated cancer cells drive tumorigenesis [10]. A recent study has shown that ropivacaine, lidocaine, and bupivacaine are effective inhibitors of leukemia stem cell colony formation and that non-cancerous stem cells are not affected by these local anesthetics [11].

GBM is a common and aggressive primary brain tumor that is usually located in the cerebral hemisphere [12, 13]. Recurrent or progressive GBM usually does not respond to standard therapy, which is associated with a poor prognosis [14, 15]. GSCs are a subset of cells that tolerate chemotherapy and radiotherapy and play a role in tumor recurrence [16–18]. Targeting GSCs and identifying new markers are the key issues involved in the development of innovative strategies to eradicate GBM [19, 20]. However, the role of local anesthetics in the growth and progression of GSCs remains unclear.

Protein s-palmitate esterification is a two-sided post-translational modification process that occurs in proteins with fatty acids and is regulated by protein acyltransferase (PAT) [21, 22]. PAT is characterized by a conserved catalytic domain containing an Asp-His-His-Cys (DHHC) motif [23, 24]. Many recent studies have shown that DHHC proteins and their substrates play a key role in tumorigenesis, particularly in the development and malignant progression of glioma [25, 26]. EZH2 palmitoylation mediated by ZDHHC5 drives malignant development and progression of GSCs [27]. ZDHHC18 and ZDHHC23 could target the GSCs of different GBM subsets in the context of their specific niches and regulate the cellular plasticity of these subtypes [28].

This study aimed to determine the possible role of local anesthetics in the self-renewal of glioblastoma stem cells and the underlying molecular mechanisms. Here, we found that local anesthetics (prilocaine, procaine, lidocaine, and ropivacaine) may impair the localization of glycoprotein 130 (GP130) through palmitoylation mediated by the zinc finger DHHC-type palmitoyltransferase 15 (ZDHHC15), inhibition of the interleukin (IL)-6/STAT3 signaling pathway, and destruction of the growth and self-renewal capacity of GSCs through a positive feedback mechanism. Our results established a direct link between palmitoylation and the inhibition effect of local anesthetics on GSCs’ growth and progression, and it could provide a significant addition to the literature on the rapid and accurate diagnosis and treatment of cancers.

## Materials and methods

### Human glioma specimens

The paraffin-embedded primary glioma tissues were obtained from the Department of Molecular Pathology, Hefei Cancer Hospital, Chinese Academy of Sciences (Anhui, China), and Department of Pathology, The First Affiliated Hospital of USTC (Anhui, China). The study (Code of Medical Ethics, Y-2018-22) was approved by the Institutional Review Committee of Hefei Cancer Hospital, Chinese Academy of Sciences, and the patient’s written informed consent was obtained, according to the Declaration of Helsinki.

### Reagents

The following local anesthetics were purchased from MedChemExpress LLC (NJ, USA): procaine (HY-B0546), dibucaine (HY-B0552), butacaine (HY-B1007), benzocaine (HY-Y0258), lidocaine (HY-B0185), oxethazaine (HY-B0955), prilocaine (HY-B0137), bupivacaine hydrochloride (HY-B0405A), ropivacaine hydrochloride (HY-B0563B), propoxycaine hydrochloride (HY-B1243), and levobupivacaine hydrochloride (HY-B0653A).

Antibodies against the following proteins were used for immunohistochemistry/immunofluorescence analysis (IHC/IF) and western blotting: ZDHHC15 (rabbit polyclonal; 1:500 for IHC/IF and 1:2000 for western blotting; Sigma-Aldrich, HPA003618), GP130 (rabbit polyclonal; 1:250 for IF and 1:1000 for western blotting; Cell Signaling Technology, #3732), STAT3 (mouse IgG2a; 1:1000 for western blotting; Cell Signaling Technology, #9139, clone 124H6), phospho-STAT3 Y705 (rabbit IgG; 1:250 for IF and 1:2000 for western blotting; Cell Signaling Technology, #9145, clone D3A7), sex-determining region Y-box (SOX) 2 (Sox2) (mouse IgG1; 1:500 for IF; Cell Signaling Technology, #4900, Clone L1D6A2), Nestin (mouse IgG1; 1:2000 for IF; Cell Signaling Technology, #33475, clone 10C2), glial fibrillary acidic protein (GFAP) (rabbit IgG; 1:500 for IF; Cell Signaling Technology, #80788, clone E4L7M), microtubule-associated protein 2 (MAP 2) (rabbit IgG; 1:500 for IF; Cell Signaling Technology, #8707, clone D5G1), and β-actin (Mouse IgG1; 1:5000 for western blotting; Sigma-Aldrich, A5441, clone AC-15).

### Cell culture

H4, A172, U87, U251, LN18, and T98G glioma cell lines were obtained between 2018 and 2020 from the Cell Bank of Type Culture Collection of the Chinese Academy of Sciences, Shanghai Institute of Cell Biology, China. They were characterized by isozyme detection and DNA fingerprinting. All cell lines were maintained at a low passage rate (5–10 passages) for experimental use and were revived every 3–4 months. The cell lines were cultured in Dulbecco’s modified Eagle’s medium (GIBCO) supplemented with 10% FBS and 1% (100×) penicillin-streptomycin (GIBCO). All cell lines used in this study were regularly verified by morphological observation and checked for mycoplasma contamination. They were last checked for mycoplasma contamination in August 2020.

The short-term in vitro amplification of GSCs was performed as previously described [28, 29]. Briefly, U87, U251, LN18, and T98G glioma cells were cultured in Thermo Fisher Scientific medium containing N2 and B27 supplements (Invitrogen), human recombinant basic fibroblast growth factor (Invitrogen), and epidermal growth factor (10 ng/ml each). Trypan blue staining followed by fluorescence-activated cell sorting (Beckman Coulter, Indianapolis, IN, USA) analysis was used to evaluate the viability of the newly formed GSC spheres, and bromodeoxyuridine (BrdU; Sigma-Aldrich, St. Louis, MO, USA) incorporation was measured to evaluate the proliferation according to the manufacturer’s instructions. Human neural stem cells (NSCs) were obtained from Lonza in 2015 and cultured in a similar manner to GSC. In order to induce the differentiation of GSCs and NSCs, the cells were cultured in the absence of growth factors or in the presence of 10% FBS (GIBCO).

### Generation and transduction of lentivirus

A lentiviral clone expressing ZDHHC15 was obtained from Origene (#TL300350V). Lentiviruses were produced in HEK293FT cells using the ViraPower Lentiviral Expression System (Invitrogen). They were concentrated by ultracentrifugation, and viral titers were determined by serial dilution.

### Luciferase assay

The pTSKL-ZDHHC15 isoform1/3 (− 681/− 837) minimal promoter was cloned by ligating the predicted STAT3 binding region (− 681/− 837) into a pTSKL plasmid between HindIII and KpnI. The pTSKL-ZDHHC15 isoform 2 (− 216/− 722) minimal promoter was similarly cloned by ligating the predicted STAT3-binding region (− 216/− 722) into a pTSKL plasmid between HindIII and KpnI.

As previously reported [30], various luciferase reporter gene constructs were transfected with Lipofectamine 2000 transfection reagent according to the manufacturer’s protocol, and the cytoplasmic fractions were prepared 48 h after transfection. In the siRNA experiments, 300 nm siRNA oligonucleotides were first added to the cells, which were then transfected with various luciferase reporter genes after 48 h, and then harvested for the luciferase assay after another 24 h. Using a colorimetric assay at A570, luciferase activity was analyzed using a microplate luminometer (Turner BioSystems) and was normalized to the β-galactosidase activity, an internal control for transfection efficiency.

### Immunoprecipitation and western blotting

For determining the protein-protein interaction, the immunoprecipitation assay was performed as previously described [31]. Briefly, cells were collected and lysed in radioimmunoassay buffer (Cell Signaling Technology, #9806) supplemented with protease inhibitors (Signaling Technology, #5872), incubated on ice for 30 min, and clarified by centrifugation at 4 °C and 12,000 rpm for 15 min. The total protein lysate (500 μg) was immunoprecipitated with agarose fixed antibody (1 μg anti-ZDHHC15) at 4 °C. SDS-PAGE and western blot were used to analyze the proteins that were immunoprecipitated and co-immunoprecipitated.

Equivalent amounts of cell lysate measured by the BCA protein assay kit (Signaling Technology, #7780) were dissolved and transferred to a polyvinylidene difluoride membrane (Millipore). It was probed with primary antibodies for 16 h at 4 °C and then blocked with 5% skimmed milk/0.1% Tween-20 in Tris-buffered saline for 1 h at room temperature. Horseradish peroxidase-conjugated secondary antibody was then used, and enhanced chemiluminescence detection (Pierce) was performed.

### Immunofluorescence analysis

For determining the self-renewal or differentiation potential, the immunofluorescence assay was performed as previously described [32, 33]. Briefly, the cells or neurospheres were fixed with 4% paraformaldehyde, washed with PBS, and incubated in a closed buffer (1× PBS containing 0.3% Triton X-100 and 5% normal goat serum) for 15 min. The cells were then incubated with primary antibodies at 4 °C for 16 h, followed by Alexa 488 goat anti-mouse (Invitrogen) and Alexa 568 goat anti-rabbit (Invitrogen) secondary antibodies. The nuclei were stained with 4,6-dimedyl-2-phenylindole (Invitrogen) and then covered with coverslips fixed with fluorescent mounting medium (Invitrogen). Images were obtained using a fluorescence microscope (IX71; Olympus), and the contrast and brightness were adjusted using the Image-Pro Plus 6.0 software (Media Cybernetics Inc., MD, USA).

### In vitro spheroidal formation assay

For the formation of suspension culture/tumorsphere, 500 cells were seeded in 6-well plates containing 2 ml of complete neurobasal medium and were either treated with local anesthetics or left untreated. After 10 days, the tumorspheres were measured and analyzed.

### Acyl-biotin exchange method

For determining the palmitoylation level, the acyl-biotinyl exchange (ABE) assay was performed as previously described [34]. Briefly, after incubating with *N*-ethylmaleimide (Thermo Fisher Scientific) to block free sulfhydryl groups on the proteins, samples were immunoprecipitated with anti-GP130 antibody (1 μg). The purified precipitates or samples were treated either with or without 1 M hydroxylamine (HAM; Thermo Fisher Scientific) and 0.5 μM BMCC biotin (Thermo Fisher Scientific) to label the palmitoylation sites. The presence of biotin on GP130 proteins was then analyzed by SDS-PAGE using horseradish peroxidase-conjugated anti-streptavidin (Cell Signaling Technology).

### Animal experiments

Animal experiments (Code of Animal Ethics, DW-2018-18) were performed following the guidelines of the Animal Use and Care Committees at the Hefei Institute of Physical Science, Chinese Academy of Sciences. All mice were randomly assigned to the appropriate treatment group. Six**-**week**-**old female C57BL/6 mice, weighing approximately 18–25 g, were anesthetized by intraperitoneal injection of ketamine (132 mg/kg) and methylthiazide (8.8 mg/kg)**,** and a cell suspension (0.1 ml) consisting of 500 U251 GSCs pretreated with local anesthetics for 5 days was subcutaneously injected on the upper left flank. After 6 weeks, the tumor**-**bearing mice were sacrificed and the tumor weights were measured.

### Statistical analysis

All grouped data are presented as the means ± standard errors. Between-group comparisons were analyzed using the Student *t* test or one-way ANOVA using GraphPad Prism version 8 (GraphPad Software, La Jolla, CA, USA). All experiments were repeated for each specimen in at least three biological duplicates. The criterion for significance (*p* values) was set as described in the figures.

## Results

### Local anesthetics impaired cell survival via downregulation of *ZDHHC15* expression

**Fig. 1:**
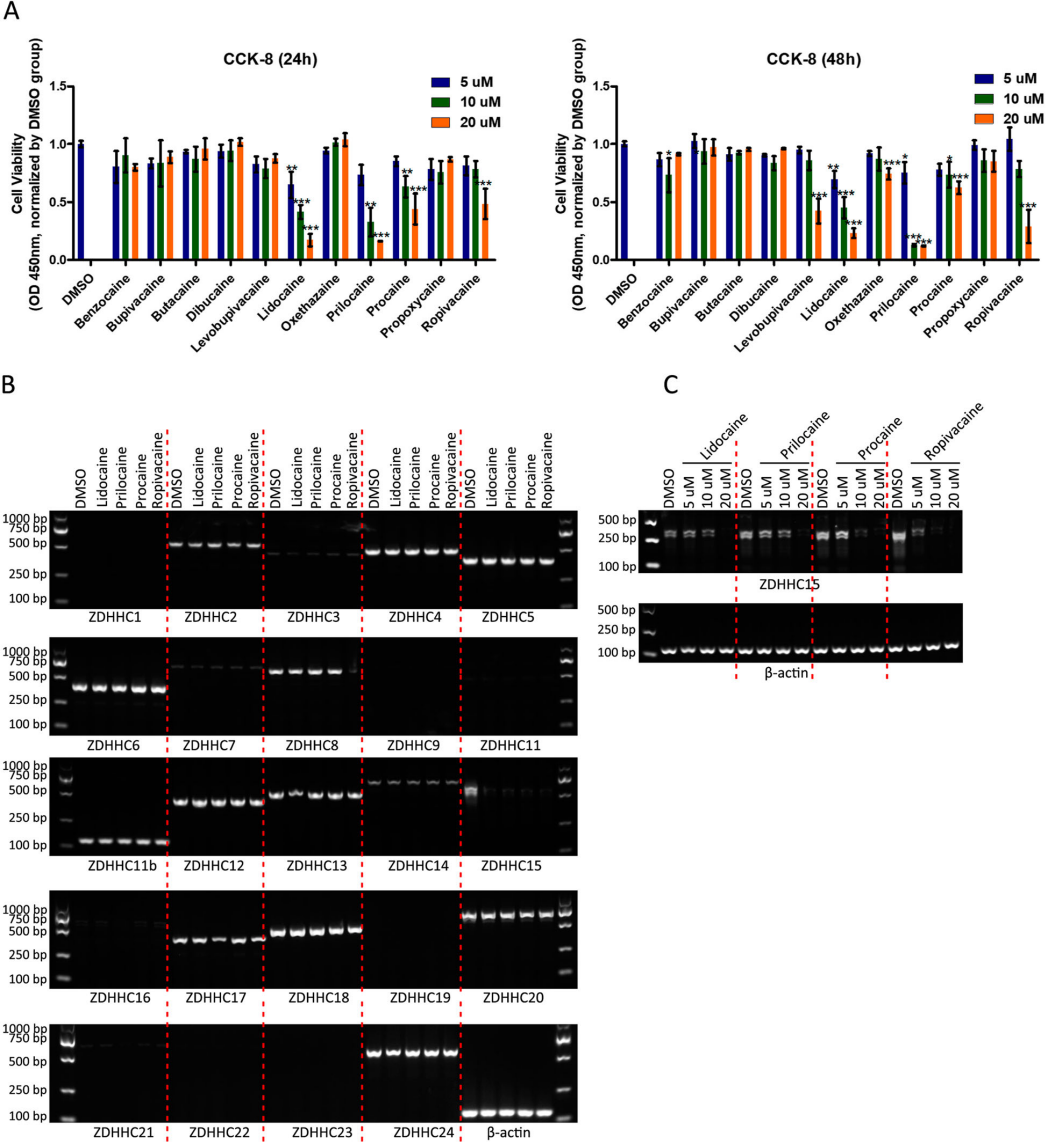
Local anesthetics prilocaine, lidocaine, procaine, and ropivacaine impaired cell survival through inhibition of *ZDHHC15* expression. Histogram showing the viability of GSCs cultured in CSC medium with or without local anesthetics (procaine, benzocaine, procaine, dibucaine, tetracaine, oxadiazine, lidocaine, propoxycaine, levobupivacaine, bupivacaine, and ropivacaine) for 24 h (left) or 48 h (right) at different concentrations (5, 10, and 20 μM, respectively). Local anesthetics, such as prilocaine, lidocaine, procaine, and ropivacaine, killed GSCs in a concentration- and time-dependent manner. **a** ART-PCR analysis of mRNA levels of 24 known pats in GSC treated with prilocaine, lidocaine, procaine, and ropivacaine (20 μm each). β-Actin was used as a loading control. **b** RT-PCR analysis of ZDHHC15 mRNA levels in GSCs treated with different concentrations (5, 10, and 20 μM) of prilocaine, lidocaine, procaine, and ropivacaine. β-Actin was used as a loading control

To study the effect of local anesthetics on malignant progression among GSCs, GSCs derived from U251 cells were subjected to in vitro treatment with 11 local anesthetics (prilocaine, benzocaine, procaine, dibucaine, butacaine, oxethazaine, lidocaine, propoxycaine, levobupivacaine, bupivacaine, and ropivacaine) after 24 h and 48 h of treatment under different concentrations (5 μM, 10 μM, and 20 μM); Cell Counting Kit 8 (CCK-8) assays were performed to determine the GSC viability (Fig. 1a). After 24 h, prilocaine, lidocaine, procaine, bupivacaine, and ropivacaine inhibited the growth of GSCs in a concentration-dependent manner. After 48 h of treatment, prilocaine, oxethazaine, lidocaine, levobupivacaine, procaine, and ropivacaine significantly suppressed GSC survival in a concentration-dependent manner. Combined with the above results, we found that the local anesthetics prilocaine, lidocaine, procaine, and ropivacaine killed GSCs in a concentration-dependent and time-dependent manner.

Palmitoylation, mediated by the DHHC family, markedly affects tumorigenesis and tumor progression through different substrates, particularly in gliomas [25, 26]. We analyzed the expression of all DHHCs in GSCs after treatment with the four local anesthetics mentioned above via reverse transcription PCR (RT-PCR) assays (Fig. 1b). Of the 24 PATs, 19 were detected in U251 GSCs, and ZDHHC1, 9, 11, 19, 22, and 23 were undetectable. ZDHHC15 was significantly downregulated in all groups treated with the local anesthetics. We also observed a significant decrease in ZDHHC15 expression levels after treatment with lidocaine, prilocaine, ropivacaine, or procaine in a concentration-dependent manner (Fig. 1c). These results indicate that the local anesthetics prilocaine, lidocaine, ropivacaine, and procaine killed cells via inhibition of ZDHHC15 expression.

### Expression pattern of ZDHHC15 isoforms in GSCs

**Fig. 2:**
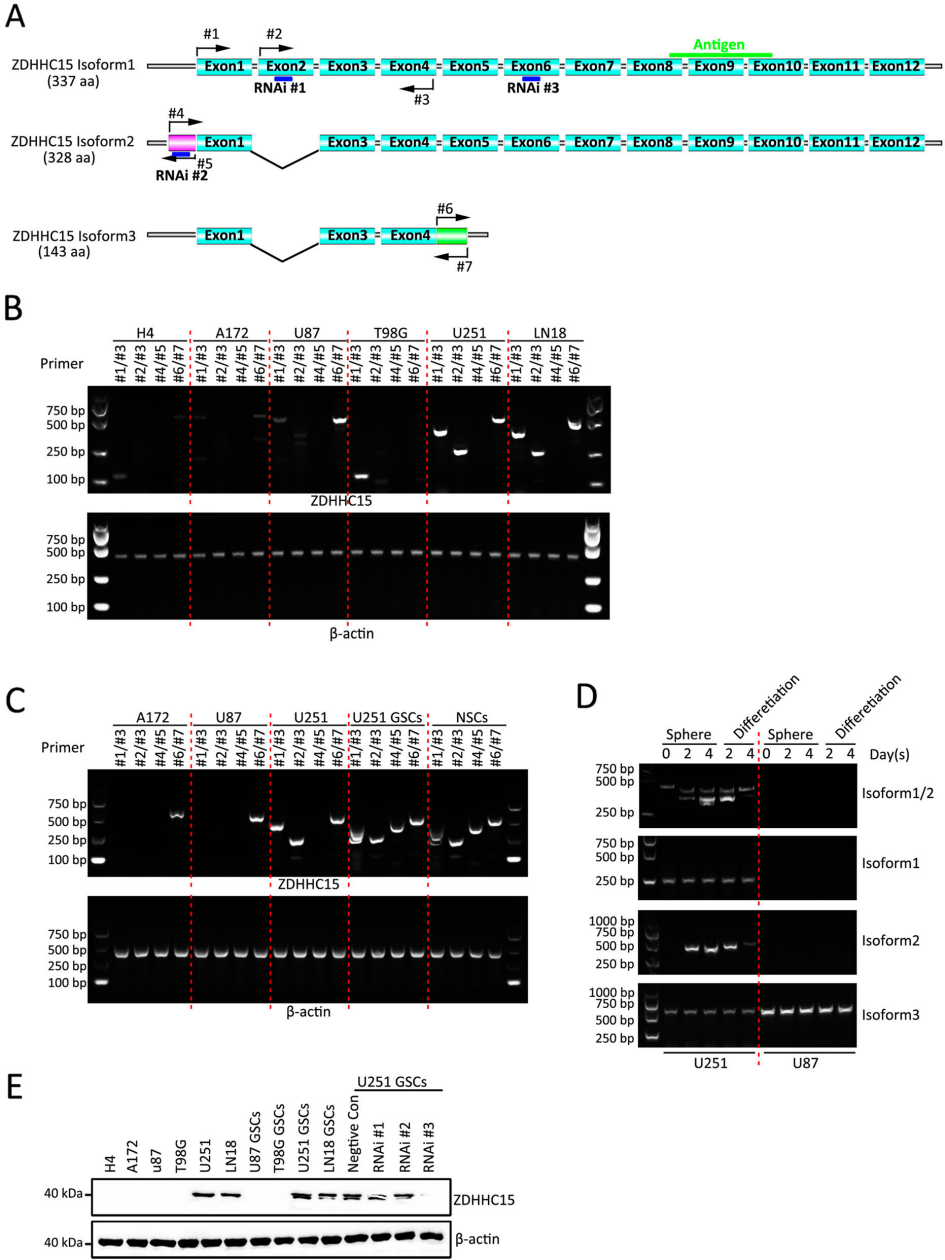
ZDHHC15 isoform expression in GBM cells and GSCs. **a** Diagram of the ZDHHC15 splicing isoforms. The isoform 1 coding sequence contains 12 exons. Compared to isoform 1, variant 2 lacks coding for exon 2, and variant 3 lacks an in-frame coding exon and differs at the 3′ end. The locations of the primers and the sites targeted with stealth siRNAs are indicated on a diagram of the ZDHHC15 splicing isoforms. **b** RT-PCR analysis of the mRNA levels of *ZDHHC15* splicing isoforms in six GBM cells (H4, A172, U87, T98G, U251, and LN18). Sequences encoding isoforms 1 and 2 using primers #1 and #3 (compared to isoform 1, variant 2 lacks 27 bp). Sequences encoding isoform 1 used primers #2 and #3. Sequences encoding isoform 2 used primers #4 and #5. Sequences encoding isoform 3 used primers #6 and #7. β-Actin served as the loading control. **c** RT-PCR analysis of the mRNA levels of *ZDHHC15* splicing isoforms in GSCs and NSCs. β-Actin served as the loading control. **d** RT-PCR analysis of the mRNA levels of *ZDHHC15* splicing isoforms during GSC self-renewal and the differentiation stage. β-Actin served as the loading control. **e** Western blot analysis of ZDHHC15 in six GBM cell lines (H4, A172, U87, T98G, U251, and LN18); four GSCs derived from U87, T98G, U251, and LN18; and U251 GSCs after transfection with ZDHHC15 stealth siRNAs. β-Actin served as the loading control

In the tumor cell lines, *ZDHHC15* has three isoforms, which are generated by alternative splicing (Fig. 2a). Transcript variant 1 encodes the longest isoform, including 12 exons. Compared with variant 1, variant 2 lacks an in-frame coding exon, resulting in a shorter isoform 2 that is missing a 9-amino acid segment. Compared with variant 1, variant 3 is missing an in-frame coding exon and differs at the 3′ end, resulting in a shorter isoform 3 with a C-terminus that is distinct from isoform 1. Transcript variant 3 does not code for protein.

First, we analyzed the expression of three isoforms of *ZDHHC15* in six human GBM cell lines via RT-PCR (Fig. 2b). Isoforms 1, 2, and 3 were undetectable in the H4, A172, and T98G cell lines. In U87 cells, only isoform 3 was detected. However, isoforms 1 and 3 were detected in U251 and LN18 cells, which belong to the classic GBM cell line (Figure S1). U87 cells have a significantly mesenchymal phenotype, and H4, A712, and T98G cells belong to the proneural subtype of cells (Figure S1). We used The Cancer Genome Atlas and Gene Expression Profiling Interactive Analysis databases and investigated the association between the expression of *ZDHHC15* and the anatomical distribution (Tables S1 and S2). The expression level of *ZDHHC15* was positively correlated with the classic GBM subtype.

Compared with monolayer cell culture, *ZDHHC15* isoform 2 could be detected in neurosphere formation, similar to that of NSCs (Fig. 2c). In particular, the expression of isoform 2 was strongly elevated during GSC self-renewal and then progressively decreased during the differentiation stage (Fig. 2d). However, isoforms 1 and 3 were not changed (Fig. 2d). Consistent with the above results, western blot analysis showed that isoform 1 was expressed in U251 and LN18 cells (Fig. 2e) and that isoform 2 could be detected in the neurospheres from U251 and LN18 cells (Fig. 2e). These results indicated that ZDHHC15 might play an important role in GBM, particularly in the classic subtype, and isoform 2 may be essential for the self-renewal of GSCs.

### Local anesthetics strongly induce differentiation and impair the self-renewal of GSCs

**Fig. 3:**
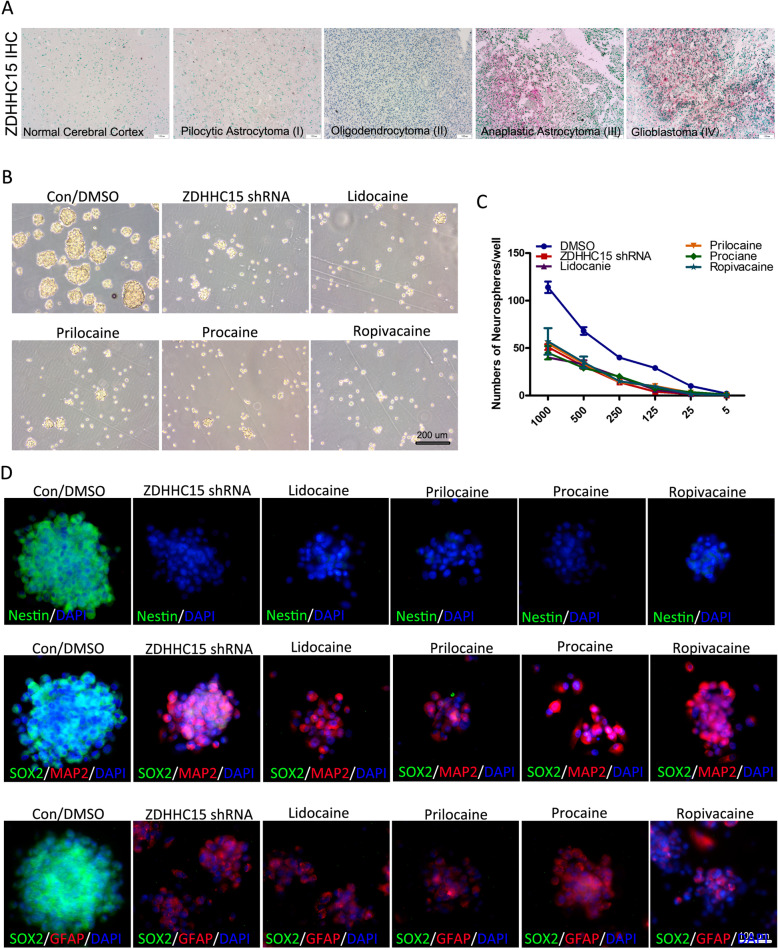
*ZDHHC15* silencing or treatment with local anesthetics strongly induces differentiation of GSCs. **a** Glioma tissue sections (*n* = 60) were stained with an antibody against ZDHHC15. Scale bar, 200 μm. **b** Representative images showing U251 GSCs maintained under neurosphere conditions for 7 days after transfection with ZDHHC15 shRNA or treatment with prilocaine, lidocaine, procaine, or ropivacaine (20 μM). **c** The capacity of the U251 GSCs transfected with ZDHHC5 shRNA and treated with prilocaine, lidocaine, procaine, and ropivacaine (20 μM) to generate neurospheres was estimated by a serial dilution assay. **d** GSC neurospheres of all categories were stained for stem and differentiated cell markers as indicated. Cells were stained with antibodies against nestin and SOX2 for neural stem cell markers, and antibody staining of GFAP and MAP 2 were used as markers of differentiated cells. DAPI (4′,6-diamidino-2-phenylindole) was used as a nuclear stain. Scale bar, 100 μm

We first compared ZDHHC15 expression in 60 glioma tissues, including pilocytic astrocytoma (PA, grade I; *n* = 6), oligodendrocytoma (OL, grade II, *n* = 18), anaplastic astrocytoma (AA, grade III; *n* = 15), and GBM (grade IV; *n* = 21), and eight normal brain tissue samples (Fig. 3a). ZDHHC15 levels in gliomas were elevated relative to the levels in the normal brain tissue and were positively correlated with the degree of malignancy. The positivity rates of ZDHHC15 were 11.11% in OL, 53.33% in AA, and 66.66% in GBM, and ZDHHC15 showed negative expression in PA.

Considering the elevated levels of ZDHHC15 in GSCs, we investigated whether ZDHHC15 is crucial for GSC self-renewal in a single cell neurosphere formation assay. shRNA-expressing lentiviruses were prepared to target ZDHHC15 expression. Under free-floating neurosphere culture conditions, *ZDHHC15* knockdown diminished the capacity of GSCs to form neurospheres (Fig. 3b). Consistent with these results, GSC maintenance was impaired by the local anesthetics procaine, prilocaine, lidocaine, or ropivacaine (20 μM each). In the serial dilution assay, U251 GSCs transduced with PBS-treated or control shRNA produced a significantly greater number of neurospheres at each level of dilution compared with the ZDHHC15-deficient cells or GSCs treated with local anesthetics (Fig. 3c). These results indicate that ZDHHC15 is required for GSC maintenance and that self-renewal of GSCs could be impaired via inhibition of *ZDHHC15* expression by the local anesthetics procaine, prilocaine, lidocaine, or ropivacaine.

The decreased formation of neurospheres in CSCs treated with procaine, prilocaine, lidocaine, or ropivacaine suggests an alteration in the “stemness” of CSCs. To provide a mechanistic insight into these phenotypic changes, we examined stem and differentiation markers in spheroids treated with the local anesthetics (Fig. 3d). Spheroids treated with the local anesthetics, namely, procaine, prilocaine, lidocaine, or ropivacaine (20 μM each), and *ZDHHC15* shRNA were stained with antibodies for several stem cells and differentiation markers. The neurosphere CSCs exhibited significant staining for nestin and SOX2, which are both neural stem cell markers, but showed limited expression of GFAP and MAP 2, both of which are markers of differentiated cells. Notably, the CSCs transfected with *ZDHHC15* shRNA or treated with the local anesthetics exhibited greatly reduced staining of nestin and SOX2, accompanied by an increased expression of the differentiated cell markers. This further confirms our findings that *ZDHHC15* silencing or local anesthetic treatment strongly induces differentiation, suggesting the existence of an additional mechanism by which ZDHHC15 inhibition could ameliorate the malignant phenotype in glioma.

### Identification of palmitoylation proteins mediated by ZDHHC15

**Fig. 4:**
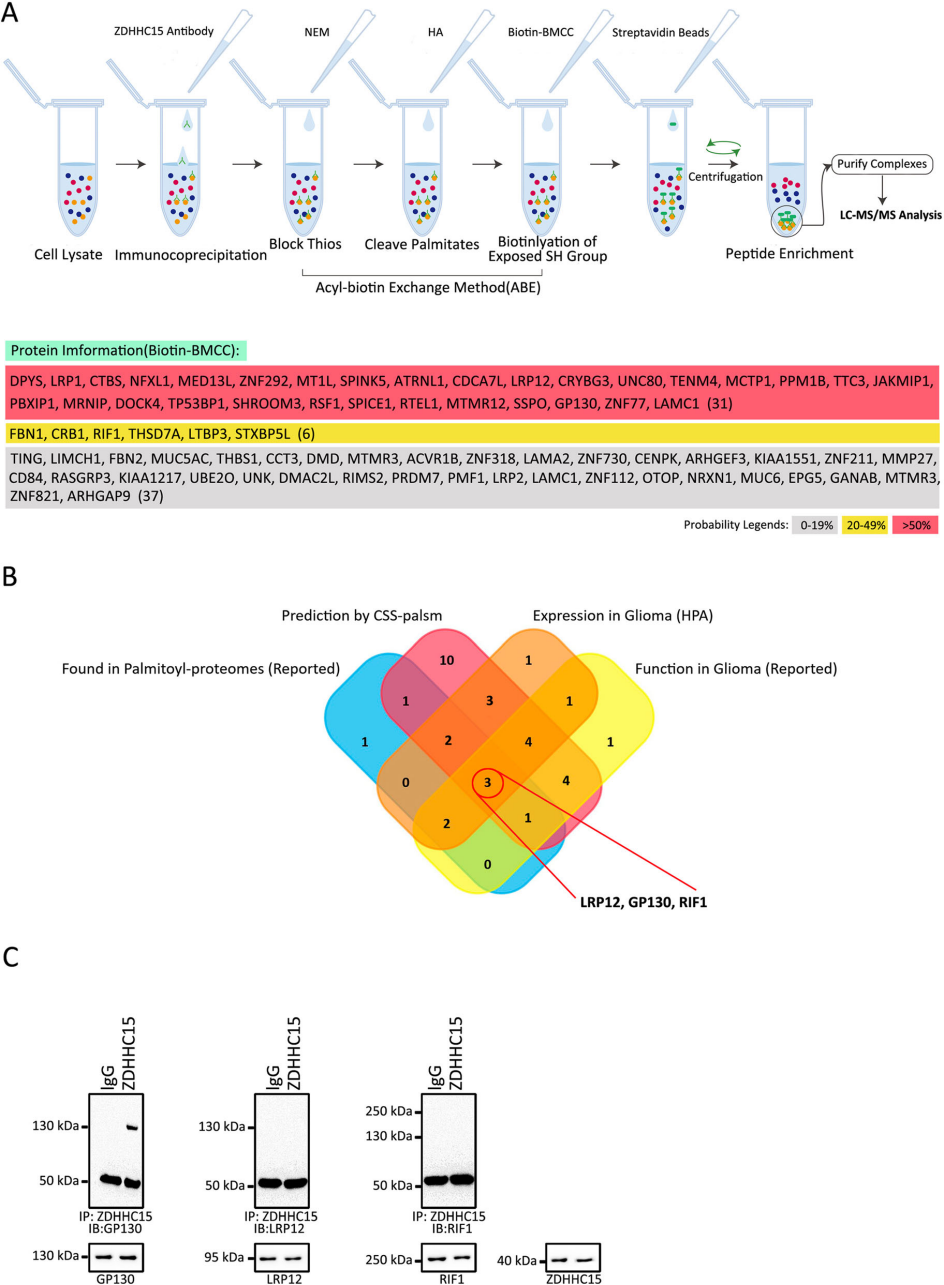
Identity of palmitoylated proteins mediated by ZDHHC15. **a** Principles of the ABE capture methods. U251 GSC was dissolved and incubated with ZDHHC15 antibody. In addition, it was cleaved with palmitate in the HAM+ group. The HAM− condition was used as a negative control. After the ABE reaction, streptavidin beads were used to enrich biotinylated proteins. The proteins enriched under HAM+ and HAM− conditions were identified by mass spectrometry (MS). In the HAM+ sample, proteins with at least 2-fold higher abundance compared to the control were considered as candidate proteins. Probability: 0–19% (2–3 times; *n* = 37), 20–49% (3–4 times; *n* = 6), and > 50% (> 4 times; *n* = 31). **b** Venn diagram showing the relationship between the expression patterns of different DHHCs in glioma using the Human Protein Atlas (HPA), previously validated S-acylated proteins, predicted S-acylated proteins using the CSS-Palm version 4.0 software, and function previously reported in glioma. **c** Lysates from U251 GSCs were subjected to IP with the ZDHHC15 antibody, followed by immunoblotting (IB) with anti-GP130, anti-LRP12, and anti-RIF1 antibodies

ZDHHC15 belongs to a super-family of PATs that catalyze the attachment of palmitate to other protein substrates [35, 36]. To identify the role of ZDHHC15 in protein palmitoylation in GSCs, we performed an ABE assay to identify proteins that are S-acylated by ZDHHC15 (Fig. 4a). U251 GSCs were lysed, incubated with the ZDHHC15 antibody, and the immunoprecipitated protein samples were divided into two fractions. In the HAM+ sample, the palmitate residue was cleaved and exchanged with biotin. The HAM− condition served as a negative control. After the ABE reaction was completed, streptavidin beads were used to enrich the biotinylated proteins. Proteins enriched under HAM+ and HAM− conditions were identified using mass spectrometry. Proteins with at least 2-fold greater abundance in the HAM+ sample were considered to be candidate proteins. Using this approach, we identified 74 palmitoylated proteins. Supporting the validity of the assay, we identified 10 previously validated S-acylated proteins, and 28 S-acylated proteins predicted using the CSS-Palm version 4.0 software (The Cuckoo Workgroup, http://csspalm.biocuckoo.org/down.php). GP130, low-density lipoprotein receptor-related protein 12 (LRP12), and Rap1 interacting factor 1 (RIF1), as candidate palmitoylated proteins for experimental validation, may be associated with glioma development and malignant progression (Fig. 4b). The results of immunoprecipitation further indicated that ZDHHC15 interacted with GP130, but not with LRP12 and RIF1 (Fig. 4c).

### Local anesthetics repressed GP130 palmitoylation and impaired its membrane localization

**Fig. 5:**
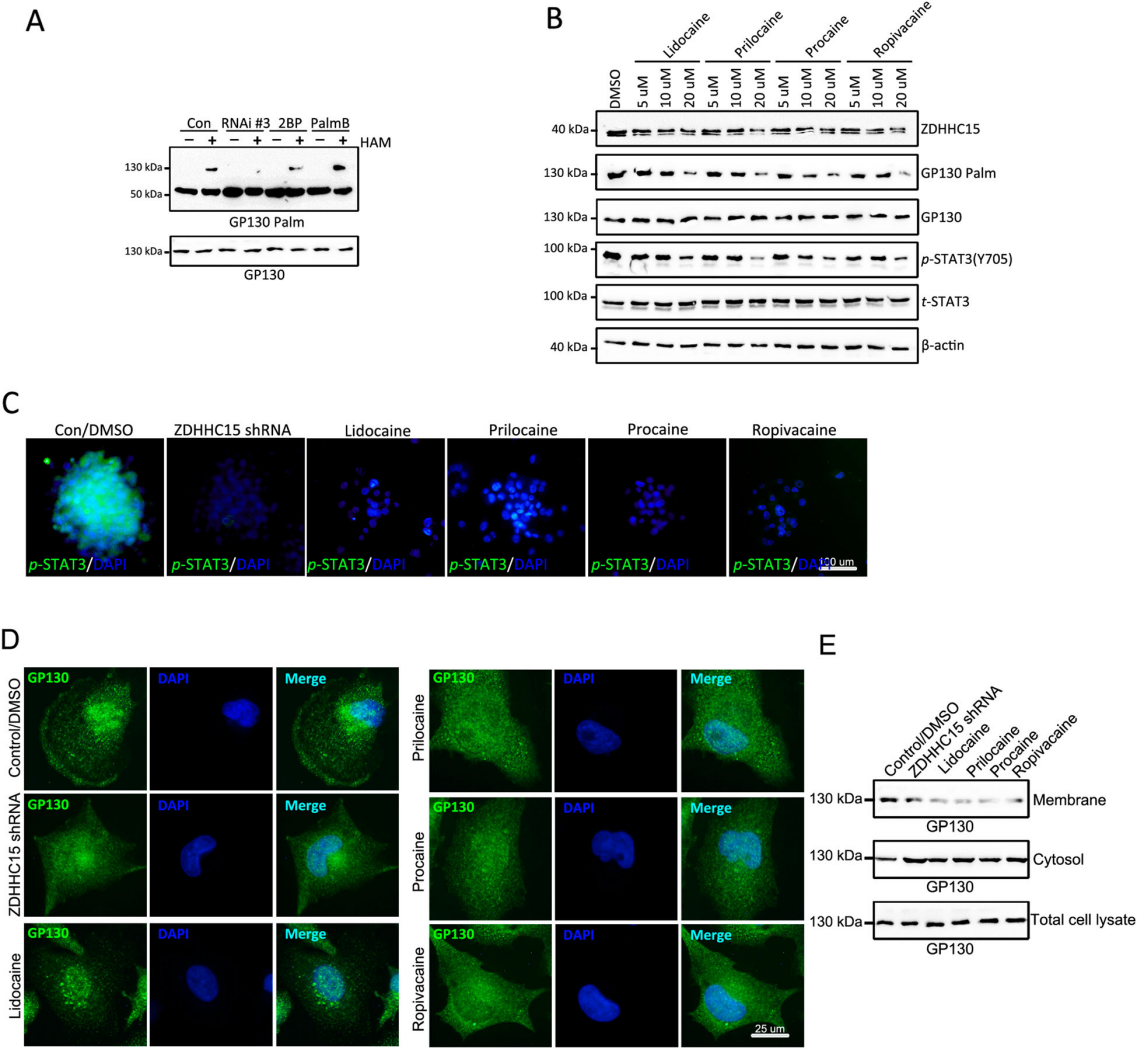
Palmitoylation inhibition mediated by the local anesthetics resulted in the disappearance of GP130 in the membrane fractions. **a** ABE was performed on proteins from U251 GSCs transfected with ZDHHC15 shRNA or treated with 2BP (50 μM) or PalmB (1 μM) for 48 h. The presence or absence of hydroxylamine (HAM) during the reaction was used as a control for reaction specificity. Western blot analysis with streptavidin-horseradish peroxidase is shown depicting the banding pattern of S-acylated proteins from 1 or 3 mg of total protein. **b** Protein accumulation (detected by western blot analysis) and palmitoylation level (detected by ABE) in GSCs treated with or without prilocaine, lidocaine, procaine, and ropivacaine at different concentrations (5 μM, 10 μM, and 20 μM, respectively). β-Actin was used as a loading control. **c** Expression of *p*-STAT3 (Y705) in U251 GSCs (monolayer culture) transfected with ZDHHC5 shRNA and treated with prilocaine, lidocaine, procaine, and ropivacaine (20 μM) for 48 h was analyzed by immunofluorescence staining. Scale bar, 100 μm. **d** The expression of GP130 in U251 GSCs (monolayer culture) transfected with ZDHHC5 shRNA and treated with prilocaine, lidocaine, procaine, and ropivacaine (20 μM) for 48 h was analyzed by immunofluorescence staining. Scale bar, 100 μm. **e** GSCs were transfected with ZDHHC5 shRNA or treated with prilocaine, lidocaine, procaine, and ropivacaine (20 μM) and harvested after 48 h. Cellular fractionation was performed to separate cytosolic and membrane fractions. Fractionates were then subjected to western blot analysis to detect the distribution of GP130

Next, we examined palmitoylation levels using an ABE assay after immunoprecipitation of GP130 and found that GP130 was palmitoylated (Fig. 5a). A significant reduction in the palmitoylation level of GP130 was observed in *ZDHHC15*-deficient or 2-bromopalmitate (2BP)-treated cells, compared with that in control cells (Fig. 5b). Moreover, we found that treatment with the depalmitoylation inhibitor palmostatin B (1 μm) resulted in the accumulation of palmitoylated GP130 (Fig. 5b). We then investigated the effects of the local anesthetics procaine, prilocaine, lidocaine, and ropivacaine on GP130 palmitoylation (Fig. 5c). In addition to the decrease in ZDHHC15 expression, the level of GP130 palmitoylation decreased after treatment with the local anesthetics in a concentration-dependent manner. Moreover, the palmitoylation status of GP130 was positively correlated with the Janus kinase/STAT3 signaling activity. Immunofluorescence results also confirmed that STAT3 (Y705) phosphorylation was decreased by *ZDHHC15* knockdown or treatment with local anesthetics (Fig. 5c). We then assessed whether GP130 palmitoylation influences its cellular distribution/localization in GSCs (Fig. 5d, e). Strikingly, we found that GSCs transfected with *ZDHHC15* shRNA or treated with local anesthetics showed reduced expression of GP130 on the membrane surface without affecting the overall expression levels, compared with normal conditions. These results indicated that palmitoylation inhibition mediated by the local anesthetics resulted in the disappearance of GP130 in the membrane fractions.

### IL-6/STAT3 regulated ZDHHC15 transcripts via positive feedback

**Fig. 6:**
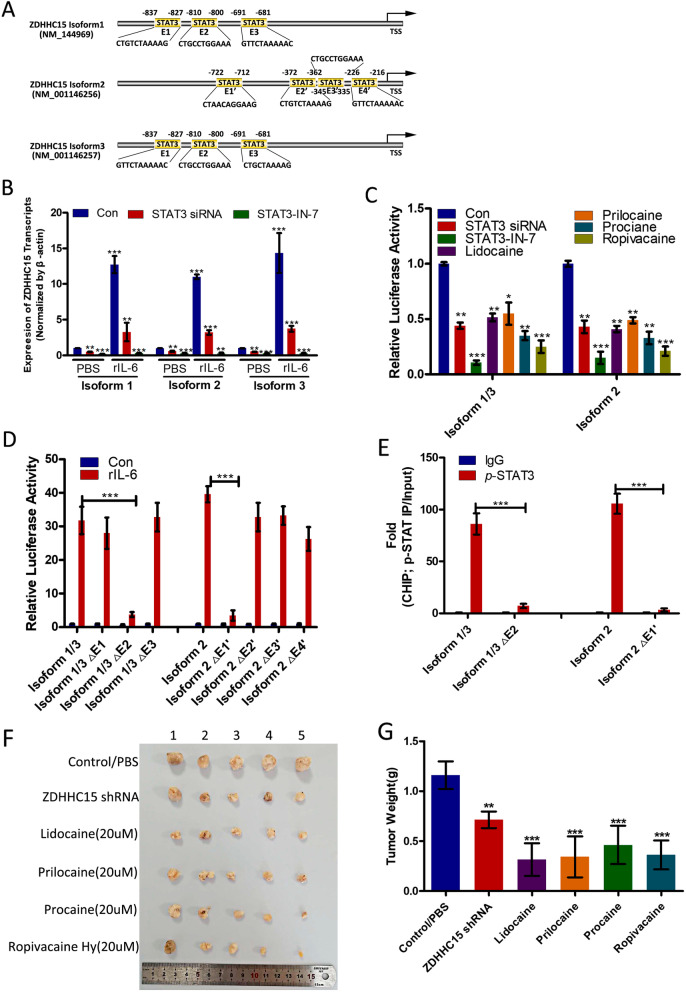
A regulatory feedback loop exists between ZDHHC15 and IL-6/STAT3 signaling. **a** Putative binding motif of transcription factor STAT3 for ZDHHC15 isoforms 1, 2, and 3 was predicted from the JASPAR database. The top three STAT3 binding sites (labeled E1′, E2, and E3) for ZDHHC15 isoforms 1 and 3 and four binding sites (labeled E1′, E2′, E3′, and E4′) were chosen for further analysis. **b** qRT-PCR was performed to evaluate the activity of STAT3 ZDHHC15 transcription in U251 GSCs transfected with STAT3 siRNA or treated with STAT3 inhibitor STAT3-IN-7 (5 μM) or rhIL-6 (5 ng/mL), alone or in combination. **c** Luciferase reporter assay demonstrated luciferase activities of ZDHHC15 isoform reporters in U251 GSCs transfected with STAT3 siRNA or treated with STAT3 inhibitor STAT3-IN-7 (5 μM), or the local anesthetics prilocaine, lidocaine, procaine, and ropivacaine (20 μM each). **d** Luciferase reporter assay demonstrated luciferase activities of various truncated reporters in U251 GSCs, determining the region of ZDHHC15 isoform promoters on which STAT3 could bind to mediate transcriptional activation. **e** ChIP assay was performed using the p-STAT3 antibody to demonstrate the enrichment of the STAT3-binding region of the promoter of ZDHHC15 isoforms in U251 GSCs. **f** Tumor growth of U251 GSCs transfected with ZDHHC5 shRNA or pretreated with prilocaine, lidocaine, procaine, and ropivacaine (20 μM). **g** Tumor weights were measured after 6 weeks (*n* = 5 mice/group)

Of note, through bioinformatics analysis with JASPAR, we discovered three putative STAT3-binding elements within the *ZDHHC15* isoform 1 and 3 promoter regions, and four STAT3 responsive elements within isoform 2 (Fig. 6a). We further investigated if IL-6/STAT3 could regulate ZDHHC15 expression in turn. STAT3 inhibitor or siRNA significantly decreased *ZDHHC15* transcription in U251 cells treated with or without rhIL-6 (Fig. 6b). A luciferase assay indicated that the *ZDHHC15* promoter was repressed by a STAT3 inhibitor or siRNA. The local anesthetics procaine, prilocaine, lidocaine, or ropivacaine could also inhibit *ZDHHC15* transcription (Fig. 6c). To clarify which element was necessary for STAT3-mediated ZDHHC15 expression, three or four predicted STAT3-binding sites were individually deleted. We found that STAT3 failed to promote *ZDHHC15* transcriptional activity without the E2 element for isoforms 1 and 3, while E1 and E3 absence alone partially downregulated *ZDHHC15* promoter activity, indicating that the E2 element was essential for STAT3 to activate transcription of *ZDHHC15* isoforms 1 and 3 (Fig. 6d). Moreover, the E1 element was essential for the activation of *ZDHHC15* isoform 2 transcription by STAT3. To further confirm these results, a chromatin immunoprecipitation (ChIP) assay was performed with *p*-STAT3 antibody, followed by detection via quantitative RT-PCR with specific primers for the E2 or E1 elements. STAT3 could associate with the *ZDHHC15* isoform 1 and 3 promoters and was enriched within the E2 region and within the E1 region of isoform 2 (Fig. 6e). These findings suggest that there is a regulatory feedback loop between ZDHHC15 and IL-6/STAT3 signaling, which may continuously activate their oncogenic functions.

To determine the efficacy of a 5-day in vitro treatment of GSCs with the local anesthetics, namely, procaine, prilocaine, lidocaine, or ropivacaine (20 μM each) on the tumor-initiating potential of GSCs, we subcutaneously injected GSCs into immunocompromised mice. Similar to the results observed with ZDHHC15 depletion, GSCs treated with local anesthetics before injection significantly suppressed tumor growth relative to control (or PBS-pretreated) animals (Fig. 6f, g). These results suggest that short-term in vitro treatment with procaine, prilocaine, lidocaine, or ropivacaine sufficiently reduced the number of tumor-initiating cells in all GSC samples, resulting in delayed tumor development.

## Discussion

Little is known about the role of local anesthetics in CSCs. This study aimed to determine the possible role of local anesthetics in GSC self-renewal and the underlying molecular mechanisms. Our data demonstrated that four commonly used local anesthetics (procaine, prilocaine, lidocaine, and ropivacaine) disrupt GSCs by targeting GP130 palmitoylation and the IL-6/STAT3 signaling pathway mediated by ZDHHC15 in a positive regulatory feedback loop.

Although early small retrospective clinical trials have shown that local anesthetics play a beneficial role in reducing tumor metastasis and recurrence in cancer patients, prospective, large-scale, and randomized clinical trials are needed to investigate the effect of regional anesthesia on long-term outcomes after cancer surgery [37]. This will confirm the significance of anesthetics for cancer patients and guide clinical practice. Local anesthetics can reach the circulatory system via absorption from the injection site or direct intravenous injection (e.g., lidocaine) to affect the circulating tumor cells released from the primary tumor during surgery [38]. Amide local anesthetics act on nerve cells by blocking voltage-gated sodium channels, resulting in decreased depolarization and repolarization rates of excitatory nerve cell membranes [39, 40]. It has been shown that common amide-linked local anesthetics exhibit anticancer activity in a variety of cancers, including lung cancer, hepatocellular carcinoma, and thyroid cancer [41, 42].

A large number of preclinical studies have shown that local anesthetics can directly inhibit the biological activities of cancer cells, including cell proliferation, migration, invasion, and survival. However, these studies were conducted using tumor cell lines representing differentiated tumor cells. Proliferation, differentiation, and self-renewal are the hallmarks of stem cells [43]. We found that four commonly used local anesthetics (prilocaine, procaine, lidocaine, and ropivacaine) significantly inhibited colony formation and self-renewal of GSCs, especially of the classic GBM subtype.

Many DHHC enzymes appear to play a key role in the tumorigenesis of glioma. Upregulation of ZDHHC5 (a carcinogen) has been reported in p53 mutant gliomas [27]. ZDHHC18 and ZDHHC23 can target specific GSCs of different GBM subsets and regulate the cellular plasticity of these subtypes [28]. In addition, the gene encoding ZDHHC17 is located in the chromosomal region containing a potential oncogene of glioma. ZDHHC17 protein can interact with MAP 2K4 to regulate the development and progression of malignant glioma and stimulate JNK/p38 [44]. In this study, we investigated the association between the DHHC family of proteins and local anesthetics in gliomas and found that ZDHHC15 was significantly downregulated in GSCs after treatment with prilocaine, procaine, lidocaine, or ropivacaine. Notably, the high expression level of ZDHHC15 is related to the classic molecular phenotype of GBM and is positively correlated with the self-renewal of GSC. Thus, it has the potential to kill the classical GBM subtypes.

Since STAT3 signal transduction is usually activated in GSCs, and its activation is necessary to maintain the self-renewal and tumorigenic potential of GSCs, destroying STAT3 signaling pathways may destroy GSCs and have a therapeutic potential [45, 46]. However, targeting the STAT3 transcription factor itself is not clinically achievable because STAT3 is essential for other functions in normal cells [47, 48]. Thus, the identification of unique upstream regulators controlling STAT3 activation in GSCs may offer new therapeutic targets for developing GSC-specific therapeutics to improve GBM treatment. In this study, we identified that ZDHHC15, a palmitoyl acyltransferase, is preferentially expressed in GSCs and demonstrated that ZDHHC15 is essential for maintaining STAT3 activation in GSCs. We found that ZDHHC15 plays a role by palmitoylation of the IL-6 receptor subunit GP130, thus promoting the activation and phosphorylation of STAT3. ZDHHC15 effectively inhibited the formation of the tumorsphere, cell proliferation, and tumor growth of GSC. As ZDHHC15 is preferentially a cell surface protein, ZDHHC15 represents a unique molecular target for the development of specific therapies for GSCs. In addition, because the expression of ZDHHC15 is positively correlated with the tumor grade of gliomas, ZDHHC15 can also be used as a useful marker for the diagnosis and prognosis of GBM.

GP130 is a glycoprotein that mediates the activation of key pro-survival pathways that are essential for tumor cell proliferation, invasion, and angiogenesis [49, 50]. Amplification of the *GP130* gene and the abnormal stabilization of the GP130 protein have been shown to be closely associated with tumor progression [51, 52]. The level of GP130 protein in normal cells is strictly regulated at the post-translational level through ubiquitin-dependent degradation, endocytosis, and caspase-induced proteolysis [53, 54]. The abnormal increase in the GP130 protein level in tumor cells may be caused by the dysregulation of post-translational processes. Shi et al. reported that tetraspanin CD9 coupled with GP130 to reduce GP130 ubiquitination, thereby sustaining high levels of GP130 in GSCs to maintain STAT3 activation [55]. In our study, we showed that GP130 is S-acylated, and its palmitoylation affects the function of the IL-6/STAT3 signaling pathway, which is crucial for many cellular processes. Post-translational modification of cellular proteins by S-acylation involves the reversible binding of fatty acids to cysteine residues, which is essential for protein transport to cell membranes and regulation of cell signal transduction. Palmitoylation is beneficial for GP130 proteins on the cell surface. However, local anesthetics such as prilocaine, procaine, lidocaine, or ropivacaine can damage ZDHHC15 transcripts and reduce the palmitoylation level of GP130 and localization to the cell membrane, thus inhibiting the activation of IL-6/STAT3 signaling.

The telomeres at the end of each chromosome will shorten when the cell divides. When the critical length is reached, the cell will enter a state of permanent cell cycle arrest, that is a state of senescence. This mechanism is thought to suppress tumors because it helps prevent precancerous cells from dividing uncontrollably. Stem cells express telomerase, which lengthens telomeres, thereby delaying aging [56, 57]. Current studies have found that palmitoylation modification mediated by the DHHC family plays an important role in DNA replication regulation, DNA damage repair, and maintenance of genome stability [58, 59]. S-acylated Rif1 mediated by palmitoyl acyltransferase Pfa4 mounts a localized DNA-damage response proximal to the inner nuclear membrane and promotes telomere homeostasis [59]. Longitudinal analyses of 147 HIV-infected participants in Baltimore between June 2010 and December 2016 suggested that cocaine use induced/accelerated telomere shortening in HIV-infected individuals [60]. Then, local anesthetics may also mediate the telomere homeostasis of cancer stem cells through ZDHHC15 or palmitoylation modification. This issue will be further explored in the future.

## Conclusions

In conclusion, our findings confirm the direct inhibitory effect of four local anesthetics on GSCs, especially the classic subtype. The potential mechanisms underlying their effect on GSCs may involve the inhibition of ZDHHC15 and its palmitoylation, inhibition of GP130 membrane localization, activation of IL-6/STAT3 signaling, and induction of GSC differentiation and damage.

## Supplementary Information

**Additional file 1: Table S1.** Co-relationship between ZDHHC15 and GBM subtype markers in The Cancer Genome Atlas database. **Table S2.** Co-relationship between ZDHHC15 and GBM subtype markers in The Gene Expression Profiling Interactive Analysis database.

**Additional file 2: Figure S1.** Heatmap showing the molecular subtype marker expression in H4, A172, U87, T98G, U251, and LN18 GBM cell lines. Proneural markers: DCX, DLL3, OLIG2, and ASCL1; mesenchymal markers: CD44, GABRA1, SLC12A5, and TIMP1; Classical markers: FOXO3, AKT2, NES, and EGFR; and Neural markers: SYT1, TGFB1, CHI3L1, and NEFL. Z-scores were calculated from the ΔCt values obtained in the qPCR analysis.

## Data Availability

All data generated or analyzed during this study are included in this published article [and its Additional files].

## Abbreviations

ABE: Acyl-biotin exchange method
CCK-8: Cell Counting Kit 8
ChIP: Chromatin immunoprecipitation
GBM: Glioblastoma
GP130: Glycoprotein 130
GSC: Glioblastoma stem cell
HAM: Hydroxylamine
LRP12: Low-density lipoprotein receptor-related protein 12
PAT: Protein acyltransferase
RT-PCR: Reverse transcription-PCR
RIF1: Rap1 interacting factor 1
ZDHHC15: Zinc finger Asp-His-His-Cys-type palmitoyltransferase 15
