# Supplementary material for: Local anesthetics impair the growth and self-renewal of glioblastoma stem cells by inhibiting ZDHHC15-mediated GP130 palmitoylation
Source: Stem Cell Res Ther. 2021 Feb 4;12:107. doi: 10.1186/s13287-021-02175-2 (PMC7863430; doi:10.1186/s13287-021-02175-2)
Supplement: Supplementary file 1 — Additional file 1: Table S1. Co-relationship between ZDHHC15 and GBM subtype markers in The Cancer Genome Atlas database. Table S2. Co-relationship between ZDHHC15 and GBM subtype markers in The Gene Expression Profiling Interactive Analysis database. [file 13287_2021_2175_MOESM1_ESM.doc]

**Table S1. Co-relationship between ZDHHC15 and GBM subtype markers in The Cancer Genome Atlas database.**

| **Correlated Gene** | **Spearman**’s Correlation | ***p***-Value |
| --- | --- | --- |
| **FOXO3** | **0.5** | **1.92E-10** |
| **SYT1** | **0.497** | **3.74E-10** |
| **AKT2** | **0.49** | **6.86E-10** |
| **NES** | **0.46** | **1.28E-08** |
| **EGFR** | **0.44** | **6.00E-08** |
| **ASCL1** | **0.44** | **3.92E-08** |
| **OLIG2** | **0.3** | **2.99E-04** |
| **CD44** | **0.25** | **2.30E-03** |
| **DLL3** | **0.2** | **0.0152** |
| **TGFB1** | **0.2** | **0.0159** |
| **DCX** | **0.195** | **0.0204** |
| SLC12A5 | 0.113 | 0.181 |
| GABRA1 | 0.0888 | 0.295 |
| CHI3L1 | 0.03 | 0.754 |
| TIMP1 | 0.02 | 0.841 |
| NEFL | -0.153 | 0.0701 |

| **GBM Subtypes** | **Classical** | **Proneual** | **Mesenchymal** | **Neural** |
| --- | --- | --- | --- | --- |

**Table S2. Co-relationship between ZDHHC15 and GBM subtype markers in The Gene Expression Profiling Interactive Analysis database.**

| **Correlated Gene** | **Spearman’s Correlation** | ***p-Value*** |
| --- | --- | --- |
| **FOXO3** | **0.43** | **1.00E-08** |
| **AKT2** | **0.42** | **3.20E-08** |
| **NES** | **0.41** | **6.40E-08** |
| **ASCL1** | **0.41** | **7.40E-08** |
| **EGFR** | **0.33** | **1.20E-05** |
| **OLIG2** | **0.31** | **4.20E-05** |
| **DCX** | **0.21** | **0.0067** |
| **CD44** | **0.18** | **0.019** |
| DLL3 | 0.15 | 0.051 |
| **GABRA1** | **0.067** | **0.4** |
| SYT1 | 0.014 | 0.86 |
| TGFB1 | −0.0022 | 0.98 |
| CHI3L1 | -0.05 | 0.52 |
| SLC12A5 | -0.12 | 0.12 |
| **NEFL** | **-0.16** | **0.047** |
| **TIMP1** | **-0.19** | **0.013** |

| **GBM Subtypes** | **Classical** | **Proneual** | **Mesenchymal** | **Neural** |
| --- | --- | --- | --- | --- |
