# Supplementary material for: Local anesthetics impair the growth and self-renewal of glioblastoma stem cells by inhibiting ZDHHC15-mediated GP130 palmitoylation
Source: Stem Cell Res Ther. 2021 Feb 4;12:107. doi: 10.1186/s13287-021-02175-2 (PMC7863430; doi:10.1186/s13287-021-02175-2)
Supplement: Supplementary file 2 — Additional file 2: Figure S1. Heatmap showing the molecular subtype marker expression in H4, A172, U87, T98G, U251, and LN18 GBM cell lines. Proneural markers: DCX, DLL3, OLIG2, and ASCL1; mesenchymal markers: CD44, GABRA1, SLC12A5, and TIMP1; Classical markers: FOXO3, AKT2, NES, and EGFR; and Neural markers: SYT1, TGFB1, CHI3L1, and NEFL. Z-scores were calculated from the ΔCt values obtained in the qPCR analysis. [file 13287_2021_2175_MOESM2_ESM.pdf]

**Figure S1**

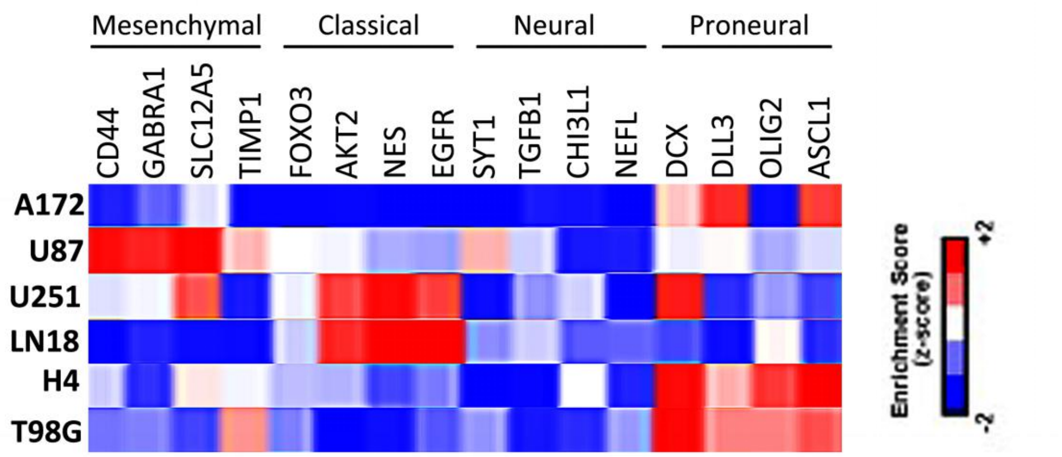


**Figure S1. Heatmap showing the molecular subtype marker expression in H4, A172, U87, T98G, U251, and LN18 GBM cell lines.** Proneural markers: DCX, DLL3, OLIG2, and ASCL1; mesenchymal markers: CD44, GABRA1, SLC12A5, and TIMP1; Classical markers: FOXO3, AKT2, NES, and EGFR; and Neural markers: SYT1, TGFB1, CHI3L1, and NEFL. Z-scores were calculated from the ΔCt values obtained in the qPCR analysis.
